# Design of parallel 𝛽‐sheet nanofibrils using Monte Carlo search, coarse‐grained simulations, and experimental testing

**DOI:** 10.1002/pro.5102

**Published:** 2024-07-22

**Authors:** Sudeep Sarma, Tarunya Rao Sudarshan, Van Nguyen, Alicia S. Robang, Xingqing Xiao, Justin V. Le, Michael E. Helmicki, Anant K. Paravastu, Carol K. Hall

**Affiliations:** ^1^ Department of Chemical and Biomolecular Engineering North Carolina State University Raleigh North Carolina USA; ^2^ Department of Chemical and Biomolecular Engineering Georgia Institute of Technology Atlanta Georgia USA; ^3^ Present address: Department of Chemistry, School of Chemistry and Chemical Engineering Hainan University Haikou City Hainan Province People's Republic of China

**Keywords:** amyloid *β*‐sheet fibrils, DMD/PRIME20 simulations, peptide assembly design, peptide self‐assembly

## Abstract

Peptide self‐assembly into amyloid fibrils provides numerous applications in drug delivery and biomedical engineering applications. We augment our previously‐established computational screening technique along with experimental biophysical characterization to discover 7‐mer peptides that self‐assemble into “parallel *β*‐sheets”, that is, *β*‐sheets with N‐terminus‐to‐C‐terminus 𝛽‐strand vectors oriented in parallel. To accomplish the desired *β*‐strand organization, we applied the *PepAD* amino acid sequence design software to the Class‐1 cross‐*β* spine defined by Sawaya et al. This molecular configuration includes two layers of parallel *β*‐sheets stacked such that N‐terminus‐to‐C‐terminus vectors are oriented antiparallel for molecules on adjacent *β*‐sheets. The first cohort of *PepAD* identified peptides were examined for their fibrillation behavior in DMD/PRIME20 simulations, and the top performing sequence was selected as a prototype for a subsequent round of sequence refinement. The two rounds of design resulted in a library of eight 7‐mer peptides. In DMD/PRIME20 simulations, five of these peptides spontaneously formed fibril‐like structures with a predominantly parallel 𝛽‐sheet arrangement, two formed fibril‐like structure with <50% in parallel 𝛽‐sheet arrangement and one remained a random coil. Among the eight candidate peptides produced by PepAD and DMD/PRIME20, five were synthesized and purified. All five assembled into amyloid fibrils composed of parallel *β*‐sheets based on Fourier transform infrared spectroscopy, circular dichroism, electron microscopy, and thioflavin‐T fluorescence spectroscopy measurements.

## 1 INTRODUCTION

We seek to establish a workflow to design previously‐unknown amino acid sequences to produce peptides that assemble into specific desired structures. It is known that peptides can self‐assemble into architectures like nanofibers (Cormier et al., 2013; Nagy‐Smith et al., 2015), nanosheets (Childers et al., 2010), nanotubes (Li et al., 2016), nanoparticles (Tian et al., 2017; Villegas et al., 2022), but understanding of the relationship between amino acid sequence and structures of assemblies is limited. Our ability to engineer supramolecular structures at the nanoscale impacts a wide variety of potential applications (Sinha et al., 2021; Wilson et al., 2018) including as polymeric biomaterials (Katyal et al., 2019), tissue‐engineering scaffolds (Cunha et al., 2011; Deidda et al., 2017; Jonnalagadda et al., 2017; Zhang, 2003), hydrogels (Boyle & Woolfson, 2011; Jonker et al., 2012; Nagy‐Smith et al., 2015; Sinha et al., 2021; Sinthuvanich et al., 2012; Woolfson, 2010), drug release agents (Altunbas et al., 2011; Branco et al., 2010), and biomineralization components (Eby et al., 2011; Mitchison et al., 2001). Short peptides are particularly desirable for biomaterials discovery because they can be easy to synthesize and, in comparison to longer peptides, they may exhibit a higher tendency to aggregate (Wilson et al., 2018). Although peptide assemblies can be composed of molecules in various secondary structures, we focus here on *β*‐sheet assemblies. “Bottom‐up” strategies, in which the amino acid sequence length, composition, and pattern are tailored, could be used to obtain supramolecular architectures with great structural variety and desired functional properties. The amino acid sequence composition of the peptides and their secondary structure drives the peptide self‐assembly process to obtain peptide‐based supramolecular assemblies.

In contrast to the “bottom up” search for amino acid sequences that we are aiming to establish here, previous designs for *β*‐sheet peptide assemblies have been inspired by fragments from naturally occurring amyloidogenic proteins, or have emphasized simple patterning of hydrophobic and polar amino acids. Examples of peptide fragments that self‐assemble into *β*‐sheet fibrils include the 7‐mer peptide fragment A*β* (16–22) (sequence: *KLVFFAE*), which is associated with Alzheimer's disease, and the fibril‐forming segment of the yeast prion protein Sup35 (sequence: GNNQQNY). Furthermore, Lynn et al. chemically modified A*β* (16–22) to assemble into nano‐sheets (Li et al., 2016; Wilson et al., 2018). Examples of peptides designed with hydrophobic/polar patterning include RADA16‐I (sequence: Ac‐RADARADARADARADA‐NH_2_) (Cormier et al., 2013; Zhang, 2002) and MAX1 (sequence: VKVKVKVKV^D^PPTKVKVKVKV‐NH_2_) (Kretsinger et al., 2005; Nagy‐Smith et al., 2015), where Ac‐ indicates an acetylated N‐terminus, –NH_2_ indicates an amidated C‐terminus, ^D^P indicates proline with D‐chirality, ‐V^D^PPT‐ corresponds to type‐II’ *β*‐turn to promote *β*‐hairpin formation.

There are eight possible classes of 2‐layer *β*‐sheet structures, called cross‐*β* structures spines, that peptides can form according to a 2007 paper by Sawaya et al. (2007). Although there have been significant advances in statistical biophysics and bioinformatics‐based tools to predict amyloidogenic regions in a peptide sequence, “bottom‐up” computational design of novel peptide sequences not known to adopt *β*‐sheet‐rich supramolecular structures is still a challenge. An early noteworthy example is a paper by Wang et al., who employed a sequence‐based QSAR approach followed by atomistic molecular dynamics simulations to design self‐assembling peptides that form A𝛽 like aggregates (Wang et al., 2014). They succeeded in designing self‐assembling hexapeptides without a preferred *β*‐strand arrangement that can be used as potential A𝛽 inhibitors in treating Alzheimer's disease.

In our previous work, we developed a workflow for computational and experimental discovery of 7‐amino‐acid peptides for self‐assembly into amyloid structures. The chosen target structure was the Class‐8 cross‐*β* spine structure described by Sawaya et al. (2007), with peptides arranged into a pair of stacked antiparallel *β*‐sheets. The workflow started with PepAD; a Monte‐Carlo‐based *pep*tide *a*ssembly *d*esign (PepAD) algorithm developed in the Hall lab. PepAD allows custom pre‐settings for design parameters, such as the peptide length, amino acid sequence, backbone scaffold, and hydration properties, to identify specific fibril‐forming peptides. Additionally, PepAD uses atomistic force‐fields rather than knowledge‐based information and hence, enables the de novo design of peptides not known in nature. The self‐assembling tendencies of the peptides identified by the PepAD algorithm are further evaluated using discontinuous molecular dynamics (DMD) simulations with the PRIME20 force field to examine their fibrilization kinetics. Eight of the 12 *in silico* peptides identified by PepAD in our previous work successfully formed fibrils in the DMD/PRIME20 simulations and self‐assembled into anti‐parallel *β*‐sheets when tested experimentally (Collier & Messersmith, 2003). Thioflavin‐T (ThT) fluorescence measurements were used to monitor amyloid fibril formation. Peptide secondary structure was probed using circular dichroism (CD) spectroscopy and Fourier‐transform infrared spectroscopy (FTIR). FTIR also reported on *β*‐strand organization within *β*‐sheets. Finally, fibrils were imaged using negative‐stain transmission electron microscopy (TEM). All peptides tested in that study exhibited nanofiber formation with FTIR signatures of antiparallel *β*‐sheets. Since the relative alignment of peptides in neighboring sheets was not examined via DMD simulation or experiment, we could not claim that these peptides should form Class 8 structures.

In this work, we sought to test the ability of our computational tools to design peptides with a different target *β*‐strand organization, the Class‐1 cross‐*β* spine defined by Sawaya et al. (2007). This structure contains a two‐layer *β*‐sheet structure, with parallel‐oriented *β*‐strands in each layer and antiparallel‐oriented *β*‐strands between the two layers. Sawaya et al. (2007) reported five peptides that form this structure, including GNNQQNY of the prion protein Sup35. We know of no designer peptides and few naturally occurring peptides in this size range that assemble into parallel *β*‐sheets. The energy associated with the hydrogen bond network of a parallel *β*‐sheet is higher than that of an antiparallel *β*‐sheet (Zhao & Wu, 2002), suggesting that antiparallel *β*‐sheets are energetically favored for peptides in this size range. Furthermore, one can use the simple heuristic that oppositely charged sidechains near opposite termini can favor antiparallel organization (Collier & Messersmith, 2003), but we know of no analogous heuristic to favor parallel *β*‐sheets. In the work described here, two rounds of designs were performed to obtain eight 7‐mer peptides that self‐assemble to form parallel *β*‐sheets. This is in contrast to our workflow for antiparallel *β*‐sheet structure, in which only one round of PepAD design followed by DMD/PRIME20 simulations was needed to produce 8 candidate parallel *β*‐sheet forming peptides for experimental testing. (As in our previous paper, the relative alignment of peptides in neighboring sheets was not analyzed.) Five of the peptides spontaneously formed fibril‐like structures with a predominantly parallel 𝛽‐sheet arrangement, two peptides formed fibril‐like structures with <50% in parallel 𝛽‐sheet arrangement, and one peptide remained as a random coil in DMD/PRIME20 simulations. FTIR, CD, electron microscopy, and ThT fluorescence spectroscopy measurements were conducted on 5 out of 8 peptides (commercially produced and received at >95% purity). These tests revealed that all 5 peptides self‐assembled into parallel *β*‐sheets.

## 2 RESULTS

### 2.1 First round of design of class 1 cross 𝛽‐spine forming peptides

PepAD is a Monte‐Carlo‐based algorithm that searches for peptide sequences that can self‐assemble to form supramolecular structures (Xiao, Robang, et al., 2022). A score function, $\Gamma_{\text{score}}$, which considers (i) the binding free energy, ${\Delta G}_{\text{binding}}$, of the peptide chain with its neighboring peptides and (ii) the intrinsic self‐aggregation propensity, $P_{\text{aggregation}}$, of the individual peptides, is used to evaluate new peptide sequences. Details are provided in Section 4.1.

**FIGURE 1:**
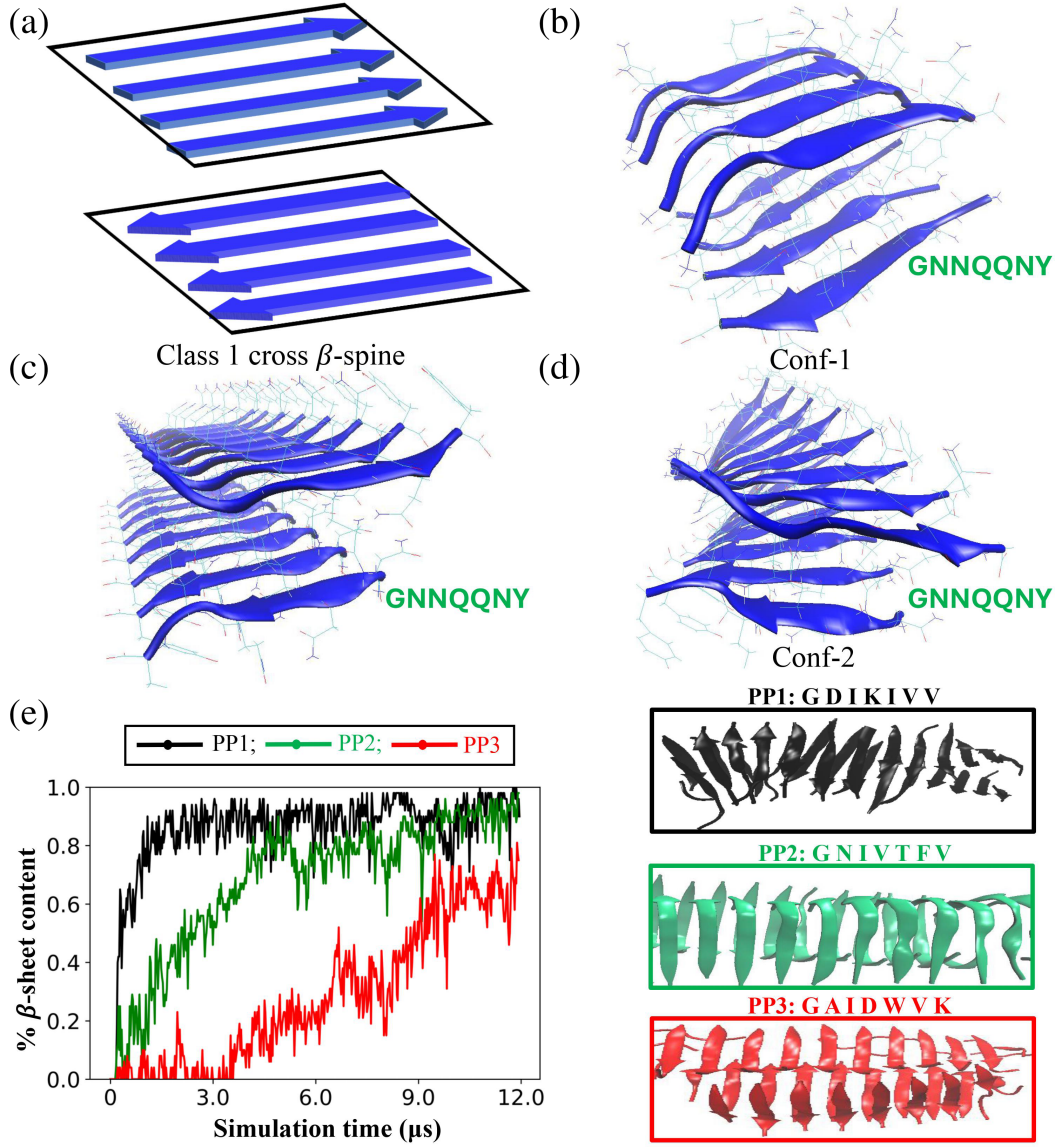
First round of design for Class 1 cross 𝛽‐spine forming peptides: (a) Arrangement of peptides in the Class 1 cross 𝛽‐spine, a two‐layer amyloid fibril consisting of parallel‐oriented *β*‐strands in each layer and antiparallel‐oriented *β*‐strands between the two layers. (b) Input fibril structure of peptide GNNQQNY for the PepAD algorithm (**Conf‐1**) constructed using Discovery Studio 3.5 and Packmol packages and optimized by atomistic MD simulation in the AMBER14 package. (c) Fibril structure of peptide GNNQQNY obtained from PDB ID: 2omm (d) Input fibril structure of peptide GNNQQNY for the PepAD algorithm (**Conf‐2**) constructed by performing a 5 ns MD simulation on structure in (c). (e) Plot of 𝛽‐sheet content versus simulation time describes the self‐aggregation kinetics of peptide PP1 (sequence: GDIKIVV), PP2 (sequence: GNIVTFV), and PP3 (sequence: GAIDWVK) (left). The snapshot of the final simulation structures of peptides PP1, PP2, and PP3 indicating their fibrilization behavior is shown (right).

The PepAD algorithm requires an initial backbone scaffold to design peptide sequences that can self‐assemble into the Class 1 cross‐𝛽 spine. As mentioned earlier, Sawaya et. al reported in their study (Sawaya et al., 2007) that the fibril‐forming segment GNNQQNY of the prion protein Sup35 forms a steric zipper characteristic of Class 1 cross‐𝛽 spine. Hence, GNNQQNY fibril was used as the reference peptide in the first round of design. This structure consists of a 2‐layer amyloid fibril whose 𝛽 strands are parallel within the 𝛽‐sheet layer and antiparallel between the 𝛽‐sheet layers (Figure 1a). Based on their study, we constructed two versions of the GNNQQNY structure that forms the Class 1 cross‐𝛽 spine; these were used as starting backbone scaffolds in two parallel design rounds. Hereafter, we refer to these two initial backbone scaffolds as **Conf‐1** and **Conf‐2**.

To obtain **Conf‐1**, we constructed a 2‐layer amyloid 𝛽‐sheet structure using the Discovery Studio 3.5 and Packmol packages. Eight 7‐mer GNNQQNY (PP0) peptide sequences were aligned in a parallel arrangement within each 𝛽 sheet layer and an antiparallel arrangement between the 𝛽 sheet layers with 4 peptides in each layer. The peptide distance within each sheet was set to be ~5.5 Å and the inter‐sheet distance was specified as ~12 Å. A 5 ns explicit‐solvent atomistic molecular dynamics simulation was conducted using the AMBER14 package to relax the two‐layer 𝛽‐sheet structure in the aforementioned parallel arrangement and to eliminate any atomic overlaps. The structure obtained following the 5 ns simulation was used as the input backbone scaffold (**Conf‐1**) for the PepAD algorithm (Figure 1b).

To build **Conf‐2**, we used the crystal structure of peptide GNNQQNY (PDB ID: 2omm) reported in the Protein Data Bank. The primary coordinate file corresponding to PDB ID: 2omm contains the crystal asymmetric unit of a single GNNQQNY sequence and provides the information needed to generate the biological assembly of four GNNQQNY peptides into a Class 1 cross‐𝛽 spine. This is a 2‐layer 𝛽‐sheet structure containing 2 parallel‐oriented 𝛽‐strands in each layer with antiparallel‐oriented 𝛽‐strands between the two layers. An in‐house Python code was written to generate a configuration of Class 1 cross‐𝛽 spine with 4 peptides (Supplementary Figure S1). Four replicas of this structure were generated to produce a two‐layer 𝛽‐sheet structure with 8 parallel‐oriented 𝛽‐strands in each layer and an antiparallel arrangement between the two layers (Figure 1c). (Code is available at: https://github.com/CarolHall‐NCSU‐CBE/Parallel‐self‐assembling‐peptides.) We performed a 5 ns explicit solvent atomistic molecular dynamics simulation using the AMBER14 package to relax the aforementioned two‐layer sheet structure to eliminate any atomic overlaps. The structure obtained following a 5 ns simulation was used as a second input backbone scaffold (**Conf‐2**) for the PepAD algorithm (Figure 1d).

Next, we specified the hydration properties for the designed amyloid‐forming peptides as input parameters for the PepAD algorithm. Two cases were investigated with two different sets of hydration properties for the peptide chain. We classify the 20 natural amino acids into four residue types: hydrophobic residues (Leu, Val, Ile, Ala, Met, Phe, Tyr, Trp, Gly), polar residues (Ser, Thr, His, Asn, Gln), charged residues (Arg, Lys, Asp, Glu) and other residues (Cys, Pro). The two cases are as follows, Case 1: *N*_hydrophobic_ = 5, *N*_polar_ = 0, *N*_charge_ = 2, *N*_other_ = 0 and Case 2: *N*_hydrophobic_ = 5, *N*_polar_ = 2, *N*_charge_ = 0, *N*_other_ = 0. (We were interested in determining which hydration properties favor the parallel amyloid‐𝛽 sheet formation.) For each case, we performed the PepAD algorithm at two different values for the weighting factor *λ*, viz. *λ* = 2.0 and *λ* = 3.0 in Equation (1). The weighting factors λ = 2.0 and λ = 3.0 were chosen to provide a good balance between optimizing the binding free energy (${\mathrm{\Delta G}}_{\text{binding}}$), and the aggregation propensity terms ($P_{\text{aggregation}}$) in the $\Gamma_{\text{score}}$ of the amyloid‐forming structure. All the searches start with random peptide sequences draped on the fixed backbone scaffold. By having random initial peptide sequences, we encourage our designs to proceed along different search pathways in sequence space and thereby sample peptides from a larger pool of peptide sequences than would otherwise be the case. The *Γ*_score_ profile fluctuates considerably as new amino acids are placed on the different sites of the peptide chain. By examining the *Γ*_score_ profiles over the sequence evolution, we identified four peptide sequences (PP1–PP4) with low scores for evaluation using DMD/PRIME20 simulations. PP1 and PP2 were identified with **Conf‐1** as the starting structure, and PP3 and PP4 were identified with **Conf‐2** as the starting structure.

We performed a preliminary screen to investigate the fibrilization kinetics of the four PepAD identified *in silico* peptides (PP1–PP4) by running DMD/PRIME20 simulations for 5μs. DMD/PRIME20 is a fast alternative to traditional molecular dynamics simulations that uses discontinuous potentials to model peptide aggregation. The force field PRIME20, developed by the Hall group in 2010, is a coarse‐grained model where each amino acid is represented by a three‐sphere backbone comprised of united atoms (NH, C_α_H, and CO) and a single‐sphere side chain, R (Bunce et al., 2021; Cheon et al., 2010; Nguyen & Hall, 2004; Wang et al., 2016; Wang et al., 2017; Wang et al., 2018). Details are provided in Section 4.2. The simulations were performed at temperatures ranging between 296 to 310 K for 5 μs. The peptides were then extensively studied at the temperature at which they showed the highest fibrillation propensities by performing 12 μs DMD/PRIME20 simulations. The simulations predict that peptide PP1 (sequence: GDIKIVV), PP2 (sequence: GNIVTFV) and PP3 (sequence: GAIDWVK) spontaneously form amyloid‐like fibrils and adopt a predominantly parallel 𝛽‐sheet arrangement. Peptide PP4 (sequence: GGIDWKI) formed amyloid‐like fibrils but exhibited less than 50% parallel 𝛽‐sheet content in the DMD/PRIME20 simulations. Table 1 contains the sequences of peptides PP1–PP4 with their associated scores, binding free energies, intrinsic self‐aggregation propensities; the number of layers in the fibrils and the parallel 𝛽‐sheet content percentage estimated from DMD/PRIME20 simulations. The % 𝛽‐sheet content versus simulation time for peptides PP1–PP3 and PP4 are shown in Figure 1e (left) and Supplementary Figure S2, respectively. (Snapshots of the final simulated structures of peptides PP1–PP3 are shown in Figure 1e (right).)

**TABLE 1:** The sequences of the four *in silico* discovered peptides in the first round of design with their corresponding *Γ*_score_, ∆*G*_binding_, and *λ* × *P*_aggregation_ values computed from PepAD.

Peptides	Case	Sequences	Γ_score_	Δ*G*_binding_	*λ* × *P*_aggregation_	DMD/PRIME20
Starting with Conf‐1						
PP1	1	GDIKIVV	−12.19	−12.37	−0.18	Two‐layer fibril, ~77% parallel 𝛽‐sheet content
PP2	2	GNIVTFV	−17.25	−12.39	4.85	Two‐layer fibril, ~100% parallel 𝛽‐sheet content
Starting with Conf‐2						
PP3	1	GAIDWVK	−8.28	−9.29	−1.01	Multi‐layer fibril, ~100% parallel 𝛽‐sheet content
PP4	1	GGIDWKI	−6.55	−6.45	0.1	Two‐layer fibril, ~46% parallel 𝛽‐sheet content

*Note*: The %Parallel 𝛽‐sheet content for each peptide is computed from the DMD/PRIME20 simulations.

### 2.2 Second round of design for Class 1 cross 𝛽‐spine forming peptides

**FIGURE 2:**
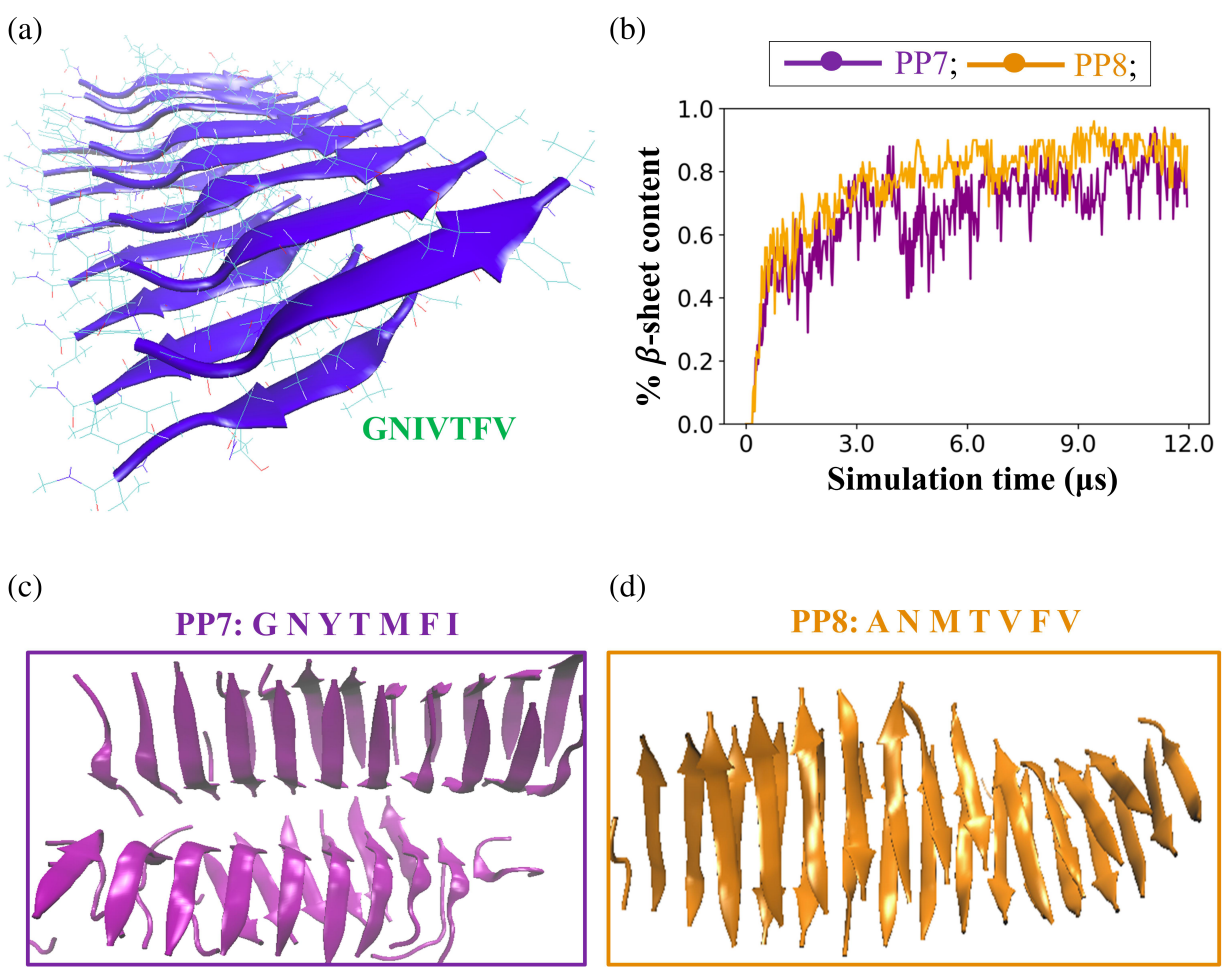
Second round of design of Class 1 cross 𝛽‐spine forming peptides: (a) Input fibril structure of peptide PP2: GNIVTFV (with patched N‐ and C‐termini) for the PepAD algorithm in Round 2 of design (**Conf‐3**). (b) Plots of 𝛽‐sheet content versus simulation time describe the self‐assembly kinetics of peptides PP7 and PP8. Snapshots of the final structures of (c) PP7 and (d) PP8 were obtained from the DMD simulations.

Since peptide PP2 (sequence: GNIVTFV) was the most promising candidate in our first round of design of Class 1 cross‐𝛽 spine forming peptides when studied via DMD simulations, it was selected as the reference sequence to create the initial backbone scaffold to perform a second round of *in silico* peptide design. (We liked that the 𝛽‐sheets that assembled in the DMD simulations were 100% parallel.) To build the backbone scaffold we used the Pymol software to mutate the residues on the Class 1 cross‐𝛽 spine structure of 16 GNNQQNY peptides (Figure 1c) to generate a Class 1 cross‐𝛽 spine structure containing 16 GNIVTFV (PP2) peptides (Supplementary Figure S3). For best comparison with the DMD/PRIME20 simulations and experimental biophysical characterization (see Section 4.3), the N‐termini and C‐termini were acetylated and amidated (N‐cap and C‐cap), respectively, in this round of design using the PepAD algorithm. (The main effect of “patching” of termini is to eliminate charges at neutral pH.) A 25 ns explicit‐solvent atomistic molecular dynamics simulation was conducted using the AMBER14 package to relax the parallel two‐layer PP2 𝛽‐sheet structure and eliminate any atomic overlaps. The structure obtained following the 25 ns simulation (Figure 2a) was used as the input backbone scaffold for the second round of PepAD design. We refer to this structure as **Conf‐3**.

We next performed the PepAD algorithm to generate a new cohort of parallel amyloid‐forming peptides with Case 1 and Case 2 hydration properties using peptide PP2 draped on **Conf‐3** as the reference peptide. Four peptides (PP5–PP8) obtained from this round were further investigated in DMD/PRIME20 simulations to study their fibrillation kinetics. We again performed a preliminary screen using DMD/PRIME20 simulations of the peptide systems for 5 μs to examine their self‐aggregation kinetics at temperatures ranging from 296.1 to 310 K. These peptides were then extensively studied at the temperatures at which they showed the highest fibrillation propensity for 12 μs in DMD/PRIME20 simulations. Our simulations revealed that peptides PP5 (sequence: ADKVMFV) and PP7 (sequence: GNYTMFI) exhibited high parallel 𝛽‐sheet content while peptide PP8 (sequence: ANMTVFV) exhibited <50% parallel 𝛽‐sheet content. Peptide PP6 (sequence: GDFVKFV) predominantly remained as random coil in our DMD/PRIME20 simulations. The sequence of peptides PP5–PP8 with their associated scores, binding free energies and intrinsic self‐aggregation propensities, and observations from DMD/PRIME20 simulations are reported in Table 2. The % 𝛽‐sheet content versus simulation time of PP7 and PP8 obtained from DMD/PRIME20 simulations are shown in Figure 2b. Snapshots of the final simulated structures of peptides PP7 and PP8 are shown in Figure 2c, d respectively. The % 𝛽‐sheet content versus simulation time for peptide PP5 is shown in Supplementary Figure S2.

**TABLE 2:** The sequences of the four *in silico* discovered peptides obtained from the second round of design with their corresponding *Γ*_score_, ∆*G*_binding_, and *λ* × *P*_aggregation_ values computed from PepAD.

Peptides	Case	Sequences	Γ_score_	Δ*G*_binding_	*λ* × *P*_aggregation_	DMD/PRIME20
Starting with Conf‐3						
PP5	1	ADKVMFV	−23.50	−23.68	−0.17	Multilayer fibril, ~87% parallel 𝛽‐sheet content
PP6	1	GDFVKFV	−26.61	−25.96	0.64	Low fibril content
PP7	2	GNYTMFI	−30.93	−27.23	3.70	Two‐layer fibrils, ~91% parallel 𝛽‐sheet content
PP8	2	ANMTVFV	−30.15	−26.99	3.16	Two‐layer fibril, ~47% parallel 𝛽‐sheet content

*Note*: The %Parallel 𝛽‐sheet content for each peptide is computed from the DMD/PRIME20 simulations.

### 2.3 Experimental evaluation of self‐assembly

We designed experiments to evaluate the effects of changing the target structure from antiparallel *β*‐sheets to parallel *β*‐sheets in the PepAD computational workflow. We employed the same experimental measurements of self‐assembly that we used previously: negative‐stain TEM imaging, FTIR, ThT fluorescence, and CD. TEM images of nanofibers provide direct observation of self‐assembly. FTIR can detect *β*‐sheets through a peak at ~1620 cm^−1^ (Cerf et al., 2009; Sarroukh et al., 2013), with some variability in the precise location. ThT fluorescence detects *β*‐sheets via dye binding and can also provide time‐dependent (kinetic) data (Krebs et al., 2005; Levine, 1993; Naiki et al., 1989). CD probes secondary structure: *β*‐strand secondary structure is an indicator of *β*‐sheet self‐assembly (Kelly et al., 2005; Woody, 1995). To facilitate comparison of different peptides, we maintained peptide concentrations for each type of measurement. We varied peptide concentrations for different techniques due to the limitations of these techniques. We performed TEM on 1 mM peptide solutions to achieve a detectable number of fibrils without too much crowding on the imaging surface. We performed FTIR at 10 mM concentrations in attenuated total reflectance mode because this measurement typically requires high peptide concentrations so that enough aggregated peptide adheres to diamond surfaces. We performed ThT fluorescence measurements at 1 mM peptide concentrations because we have previously observed tractable aggregation kinetics at this concentration. We performed CD at 0.2 mM to avoid noise due to light scattering when fibril abundances are too high. When employed at this low concentration, CD can also reveal differences in peptide tendencies to form *β*‐sheets.

Of central importance in this study is the ability of FTIR to also differentiate between parallel and antiparallel *β*‐sheets via the “*β*‐Sheet organizational index” or “*β*‐index” defined by Celej et al. to be the ratio of the intensity of the peak between 1693 and 1697 cm^−1^ to the intensity of the peak between 1624 and 1632 cm^−1^ (Cerf et al., 2009; Celej et al., 2012; Hubin et al., 2015; Sarroukh et al., 2013). Celej et al. assigned *β*‐index values under 0.1 to parallel *β*‐sheets and values above 0.1 to antiparallel *β*‐sheets. Hubin et al. interpreted *β*‐index values similarly for fibrils of the Alzheimer's amyloid‐*β* peptides and oligomeric intermediates, which can be organized into parallel or antiparallel *β*‐sheets, depending on the specific aggregate (Hubin et al., 2015).

We experimentally evaluated the assembly of 5 out of the 8 candidates in Tables 1 and 2. We ordered commercial production of PP1, PP2, PP3, PP4, PP7, and PP8 (note that PP8 exhibited low fibrillar content in the DMD/PRIME20 simulations), but PP2 was not experimentally tested as it did not meet purity standards of 95%. Peptides PP5 and PP6 were not synthesized. All five final sequences formed parallel 𝛽‐sheet nanofibers as evidenced by biophysical characterization techniques.

**FIGURE 3:**
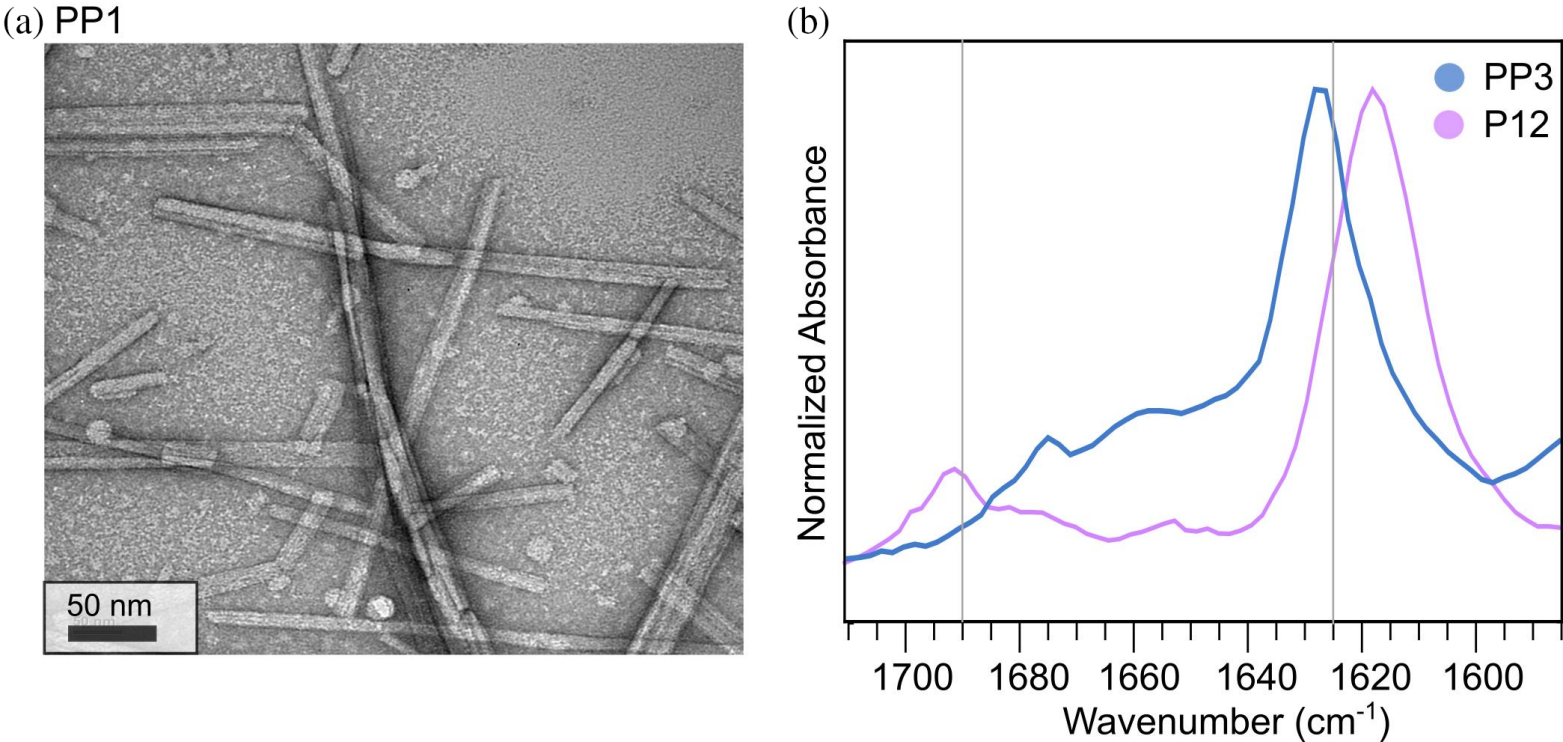
TEM imaging and FTIR spectra (a) TEM image of PP1 (b) FTIR spectra comparing P12 from our previous work and PP3. Note that PP3 shows an absence of a peak at 1690 cm^−1^, which corresponds to antiparallel *β*‐sheets.

**FIGURE 4:**
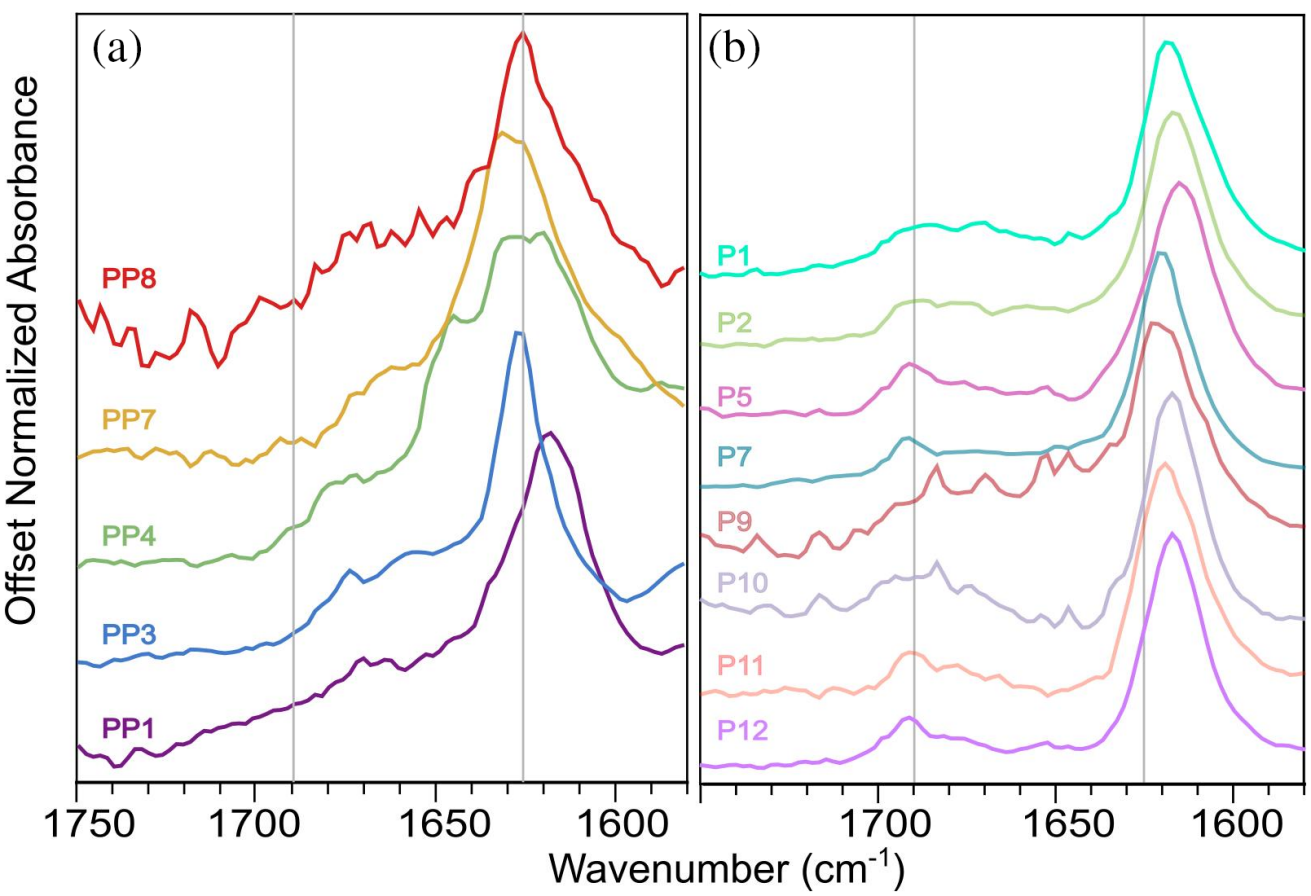
FTIR spectra comparing peptides in our present study (left) with peptides from our previous study (right). Vertical lines are drawn at the approximate positions of expected peaks for all *β*‐sheets (1625 cm^−1^) and antiparallel *β*‐sheets (1690 cm^−1^). Note that the peak at 1690 cm^−1^, associated with antiparallel *β*‐sheets, appears absent in spectra on the left.

**FIGURE 5:**
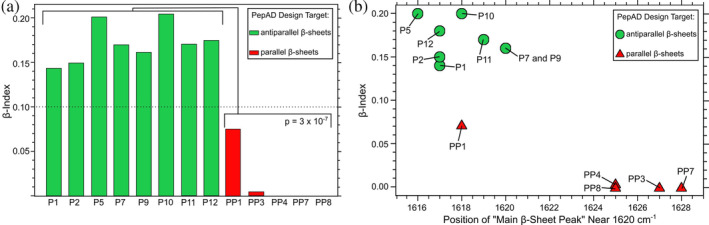
(A) Bar chart comparing *β*‐indices for peptides designed to form antiparallel *β*‐sheets (green) to those designed to assemble into parallel *β*‐sheets (red). The 1‐tail Student's *t*‐test indicates a clear difference between the population means. The horizontal dotted line indicates the threshold value of 0.10: Celej et al. interpreted *β*‐index values above and below this number to antiparallel and parallel *β*‐sheets, respectively.

We observed the most definitive evidence of assembly and parallel 𝛽‐sheet formation using TEM and FTIR. Figure 3a is a TEM image of fibrils of peptide PP3 nanofibers. Supplementary Figure S4 shows TEM images of all peptides in this series. TEM images reveal that all peptides we tested form fibrils with thicknesses consistent with multi‐layer 𝛽‐sheets. FTIR is sensitive to *β*‐sheet secondary structure and can differentiate between parallel and antiparallel *β*‐sheet structures. An FTIR peak near 1620 cm^−1^ indicates *β*‐strand secondary structure; following the nomenclature of Saroukh et al., we call this the “main *β*‐sheet peak” for *β*‐strands (Sarroukh et al., 2013). An additional peak near 1690 cm^−1^ is attributed to antiparallel *β*‐sheets (Cerf et al., 2009; Sarroukh et al., 2013). Figure 3b compares the FTIR spectra from peptide PP3 to peptide P12 (sequence: ALRLELA) (Collier & Messersmith, 2003). P12 is a peptide from our previous effort to design peptides to form antiparallel *β*‐sheets. As expected, the spectra of both peptides include main *β*‐sheet peaks near 1620 cm^−1^ (1619 and 1625 cm^−1^, respectively) indicating 𝛽‐strand secondary structure. The additional peak at 1690 cm^−1^, observed for P12 but not PP3, is associated with anti‐parallel organization of 𝛽‐sheets. Figure 4 compares FTIR spectra from all peptides tested in our present work (Figure 4a) to all peptides tested in our previous work (Figure 4b) (Collier & Messersmith, 2003). The former group of peptides exhibited no peak (or weak signal) near 1690 cm^−1^, whereas the latter group of peptides did exhibit distinct peaks near 1690 cm^−1^. To measure the *β*‐index values from the FTIR spectra, we flattened the baseline of each spectrum, performed data smoothing, and fit peaks to Gaussian functions (see Supplementary Figures S5–S7). Figure 5a visualizes the peptide‐to‐peptide variation in *β*‐index with a bar chart. As anticipated by Celej et al., *β*‐index values are above 0.1 for the peptides designed to assemble into antiparallel *β*‐sheets, and *β*‐index values are under 0.1 for the peptides designed to assemble into parallel *β*‐sheets. We confirmed the difference in *β*‐index between the two groups of peptide using an independent 2 sample 1‐tail *t*‐test (Figure 5a; significance, *p* = 3 × 10^−7^). We also compared the precise frequencies of main *β*‐sheet peak between the two groups of peptides (designed to form antiparallel vs. parallel *β*‐sheets). A 1‐tail *t*‐test revealed that the peptides designed to form parallel *β*‐sheets have main *β*‐sheet peak signals at higher frequencies, on average, than the peptides designed to form antiparallel *β*‐sheets (significance, *p* = 5 × 10^−4^). Figure 5b visualizes the correlation between main *β*‐sheet peak frequency and *β*‐index, suggesting that both parameters could be used together to assign *β*‐strand organizations to parallel or antiparallel. Overall, we interpret these results to indicate that our design workflow was successful in producing self‐assembling peptides with the desired parallel 𝛽‐strand organization. As discussed subsequently, the peptide PP1 appears to be an outlier in Figure 5b.

**FIGURE 6:**
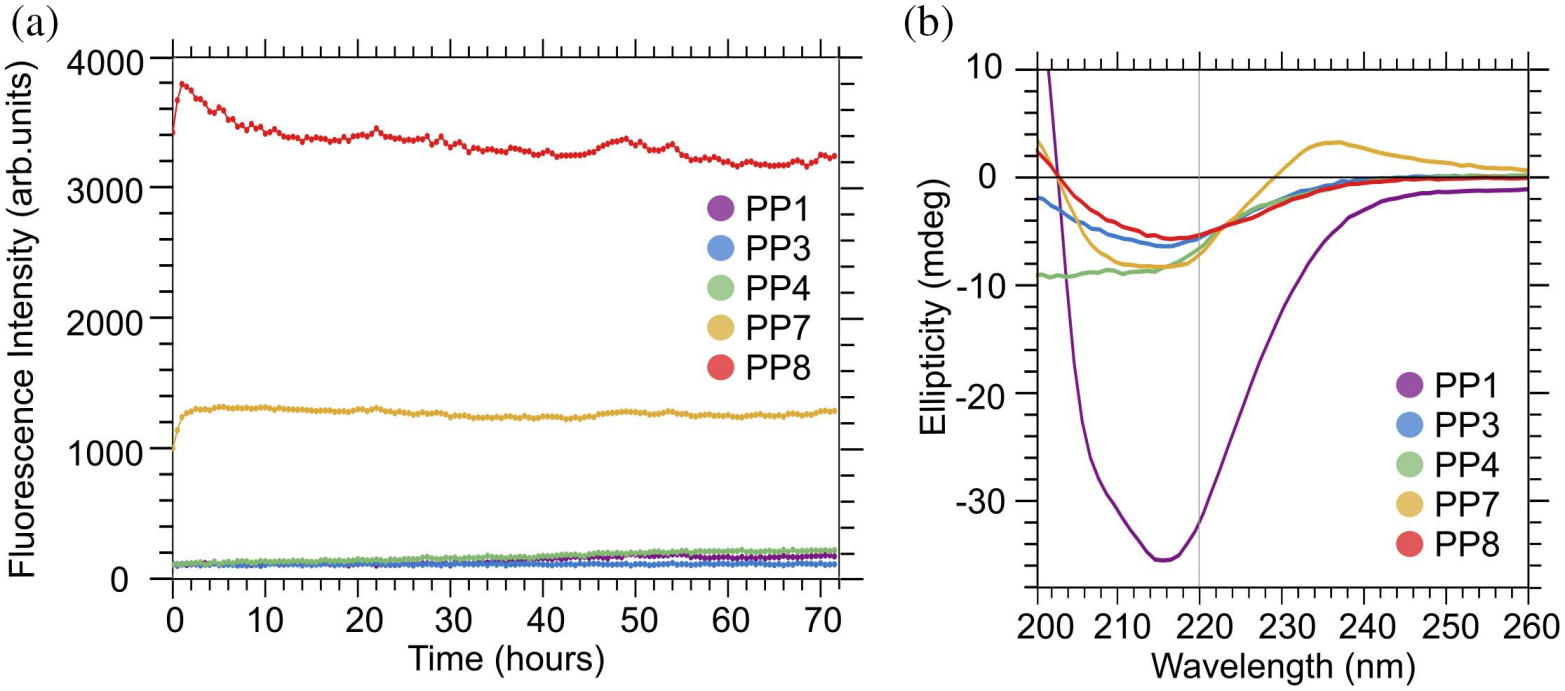
Thioflavin‐T fluorescence and circular dichroism. (a) Fluorescence intensity plotted with time for peptides in our present study. (b) Circular dichroism experiments indicate that there is a dip at ~215 nm characteristic of *β*‐sheet secondary structure.

We used ThT fluorescence measurements to probe kinetics of *β*‐sheet formation at peptide concentrations of 1 mM. Figure 6a indicates that PP7 and PP8 assemble immediately, while PP4 and PP1 show slow but increasing levels of fluorescence over 72 h. PP3 has low fluorescence levels but shows evidence of assembly through other tested experimental methods. Note that the absolute fluorescence level cannot be readily compared between different peptides: they are affected by factors such as binding of ThT to *β*‐sheet surfaces and conformation of bound ThT molecules, which both depend on amino acid sequence. There are differences in the kinetics of assembly among these peptides. PP4 shows a gradual increase in fluorescence during ThT experiments indicating slower assembly than PP7 and PP8 (see Supplementary Figure S8). In fact, definitive FTIR spectra for PP3 and PP4 were obtained only after 6 days post‐assembly. In contrast, PP7 and PP8 assemble immediately during ThT fluorescence experiments. This variation in kinetics was not observed in our previous study of peptides that form antiparallel 𝛽‐sheets.

We performed CD experiments on the peptides to characterize their secondary structure at the low concentration of 0.2 mM. Assembled 𝛽‐sheets are expected to show a single minimum near 220 nm in CD spectra, which is consistent with most of the spectra in Figure 6b. Some of the spectra exhibited evidence of conformational heterogeneity, including a shift in the local minimum below 220 nm for PP7 and PP1, and a local maximum at 235 nm for PP7. The spectrum of PP4 does not exhibit a local minimum, indicating a random coil structure.

To summarize, the five sequences designed by the computational algorithm produced assemblies that exhibit parallel *β*‐strand organization. TEM and ThT results confirmed the presence of *β*‐sheets. CD results encompass partially formed *β*‐sheets owing to lower concentration and lower assembly time periods. FTIR results show the relative decrease in the antiparallel signature for the peptides designed to form parallel *β*‐sheets. Table 3 summarizes the results of DMD/PRIME20 simulations and experimental measurements (FTIR, ThT, TEM, and CD) of peptides PP1–PP8.

**TABLE 3:** Summary of computational analysis (DMD/PRIME20) and experimental measurements (FTIR, ThT, TEM, and CD) of peptides PP1–PP8.

Peptide	DMD/PRIME20 simulation	FTIR (peptide concentration 10 mM)	ThT (peptide concentration 1 mM)	TEM (peptide concentration 1 mM)	CD (peptide concentration 0.2 mM)
PP1	Two‐layer fibril, ~77% parallel 𝛽‐sheet content (fast aggregation)	𝛽‐sheet, 𝛽‐index: 0.072	𝛽‐sheet content (slow aggregation)	Multilayer fibrils	𝛽‐sheet
PP2	Two‐layer fibril, ~100% parallel 𝛽‐sheet content (delayed aggregation)	Could not be synthesized at required purity	Could not be synthesized at required purity	Could not be synthesized at required purity	Could not be synthesized at required purity
PP3	Multi‐layer fibril, ~100% parallel 𝛽‐sheet content (slow aggregation)	𝛽‐sheet, 𝛽‐index: 0	No fluorescence observed	Multilayer fibrils	𝛽‐sheet + random coil
PP4	Two‐layer fibril, ~46% parallel 𝛽‐sheet content (slow aggregation)	𝛽‐sheet, 𝛽‐index: 0.004	𝛽‐sheet content (slow aggregation)	Multilayer fibrils	𝛽‐sheet + random coil
PP5	Multilayer fibril, ~87% parallel 𝛽‐sheet content (delayed aggregation)	Not synthesized	Not synthesized	Not synthesized	Not synthesized
PP6	Low fibril content	Not synthesized	Not synthesized	Not synthesized	Not synthesized
PP7	Two‐layer fibrils, ~91% parallel 𝛽‐sheet content (fast aggregation)	𝛽‐sheet, 𝛽‐index: 0	𝛽‐sheet content (fast aggregation)	Multilayer fibrils	𝛽‐sheet + random coil
PP8	Two‐layer fibril, ~47% parallel 𝛽‐sheet content (fast aggregation)	𝛽‐sheet, 𝛽‐index: 0	𝛽‐sheet content (fast aggregation)	Multilayer fibrils	𝛽‐sheet + random coil

## 3 DISCUSSION AND CONCLUSION

The goal of this work was to identify peptides that self‐assemble to form fibrils composed of parallel *β*‐sheets. Inspiration for this work was the Class‐1 cross‐*β* spine structure described by Sawaya et al. (2007). that contains two *β*‐sheet layers parallel‐oriented *β*‐strands in each layer, and antiparallel‐oriented *β*‐sheets between the two layers. Thus far, only one 7‐mer peptide, the fibril‐forming segment GNNQQNY of the yeast prion protein Sup35, has been identified in the literature as forming amyloid fibrils of the 1st class in experiments. As a first step toward discovering new Class‐1 peptides, we set out to design peptides that form fibrils with parallel *β*‐sheets, regardless of the relative orientation of peptides in neighboring layers, by augmenting the workflow involving PepAD, DMD/PRIME20 simulations, and experimental characterization. By using the PepAD algorithm coupled with DMD/PRIME20 simulations, we performed two rounds of designs with three different starting backbone scaffolds (**Conf‐1**, **Conf‐2,** and **Conf‐3**) to obtain a library of 7‐mer amyloid‐forming peptides that could potentially assemble into parallel *β*‐sheets. This work complements our previous study where we identified peptides that assemble into anti‐parallel *β*‐sheets as are found in the cross‐*β* spine of the 8th class. DMD simulations with the PRIME20 force field helped us computationally analyze the self‐aggregation kinetics of the peptides identified by PepAD. Five out of the eight peptides were synthesized and experimentally tested, and all of them aggregated to form parallel *β*‐sheets. Experimentally, aggregation was detected by observation of nanofibers in TEM images, measurement of CD curves consistent with *β*‐stand secondary structure, positive ThT binding as detected by fluorescence, and FTIR spectra reporting parallel organization of *β*‐sheets. The primary focus of experimental evaluation is to assess the computational workflow's efficacy in designing sequences for the target structure, specifically, an antiparallel *β*‐sheet or a parallel *β*‐sheet. Our study has successfully achieved this objective: the peptides designed for the present study exhibited lower *β*‐index value in FTIR spectra than the peptides from our previous study.

Although we were successful in showing that computational designs can control organization of *β*‐strands within *β*‐sheets, there are experimental observations that are not readily interpretable based on outputs of PepAD and DMD/PRIME20:

CD and FTIR data suggest that the designed peptide assemblies may not be perfectly homogeneous structurally. The CD data (Figure 5b) exhibit curves that do not perfectly match expectations for *β*‐sheets, though this behavior may be a consequence of the low peptide concentration we employed for CD. Regarding the FTIR data, we observed considerable peptide‐to‐peptide variation in *β*‐index, suggesting that assemblies may have included mixtures of parallel and antiparallel *β*‐sheets. We suggest that the peptide PP1 may contain a mixture of antiparallel and parallel *β*‐sheets since it appears to be an outlier in Figure 4c. In addition, as shown in Figure 4 and Supplementary Figures S5–S7, the amide I regions of some FTIR spectra included peaks other than the frequencies considered in the *β*‐index. These FTIR peaks have been attributed to random‐coil structures (1660–1670 cm^−1^), α‐helices (1650–1660 cm^−1^), and low‐molecular‐weight *β*‐sheet aggregates (oligomers: 1635–1650 cm^−1^) (Celej et al., 2012; Cerf et al., 2009; Guo & Wang, 2012). Nevertheless, the observed systematic change in *β*‐index values in the current study when compared to the previous study (Figure 4b) shows that modifying the computational workflow with a new target can affect the *β*‐strand organization.

ThT measurements indicate a large peptide‐to‐peptide variation in assembly kinetics, with PP7 and PP8 exhibiting maximal ThT fluorescence in ~1–2 h and PP3 and PP4 showing continuous increase in ThT fluorescence intensity over the course of 72 h. The assembly kinetics from the DMD/PRIME20 simulations, the % 𝛽‐sheet content versus simulation time (in μs), for the different peptides did not correlate with experimental observations. For future work, we hope to conduct a detailed study to quantify aggregation rates based on the peptide sequences and compare these to experimental results. The fibril formation time scales can be related to the population of aggregation‐prone conformations within the monomer ensemble, as Li et al. 2010, Chakraborty et al., 2020; have shown, based on coarse‐grained monomer simulations. In a series of papers, they argued that structures that have high propensity to aggregate are encoded as high‐free energy states in the monomer‐free energy spectrum. Although the nanofiber thicknesses varied (Supplementary Figure S4), all the observed thicknesses were far larger than the expected dimension based on 2 *β*‐sheet layers. (The interlayer distance between the two sheets from atomistic molecular dynamics simulations is ~4‐5 Å.) Therefore, all of the peptides observed to assemble formed *β*‐sheet interfaces that were not anticipated in the PepAD algorithm.

To summarize, we now have a computational workflow, PepAD algorithm & DMD/PRIME20, which can output novel sequences that form a desired organization of *β*‐sheets. Thus far we have succeeded in designing peptides that control assembly into parallel or antiparallel *β*‐sheet structures. The predicted structures have been experimentally tested in our previous and current work. For our future work, we could experimentally probe the stacking of *β*‐sheets to validate the predictions of the computational workflow. Additionally, the computational workflow could be developed to discourage the multi‐layer fibril formation observed experimentally.

## 4 METHODS

### 4.1 Peptide assembly design algorithm

The PepAD algorithm is a Monte Carlo (MC)‐based search procedure to discover peptides that can self‐assemble to form supramolecular architectures. In this work, we have focused on designing peptides that self‐assemble into parallel peptides. The procedure is described briefly below.

(1) *Generate input peptide backbone scaffold*: A backbone scaffold of a reference peptide is required to start the design process. In this work, a peptide scaffold corresponding to the Class 1 cross *β*‐spine (two‐layer 𝛽‐sheet structure with 2 parallel‐oriented 𝛽‐strands in each layer and antiparallel oriented 𝛽‐strands between the two layers) is generated.

(2) *Compute score of initial peptide backbone scaffold*: The tendency of the initial peptide backbone scaffold to self‐aggregate into a well‐organized amyloid‐like structure is evaluated using, *Γ*_score_, a score function that considers the binding affinities between the neighboring chains on the peptide backbone scaffold (${\mathrm{\Delta G}}_{\text{binding}}$), and the intrinsic aggregation propensities of the individual peptides ($P_{\text{aggregation}}$). The *Γ*_score_ is defined to be as follows:

(1) $$\Gamma_{\text{score}}={\mathrm{\Delta G}}_{\text{binding}}-\lambda \times P_{\text{aggregation},}.$$

where *λ* is a weighting factor that adjusts the relative importance of the intrinsic aggregation propensity of the peptides during the sequence evolution.

(3) *Iteration of peptide sequence change moves*: The PepAD algorithm performs 10,000 evolution steps and generates variants of the reference peptide by performing two kinds of trial moves, viz. (i) *residue mutation* in which an old residue on all of the peptide chains is randomly chosen and replaced by a new one of the same residue type (hydrophobic, polar, charge, and other); (ii) *residue exchange* in which two residues on all of the peptide chains are randomly chosen and swapped, regardless of their residue type.

(4) *Evaluate score* $\Gamma_{\text{score}}$ *of new peptide sequence*: The *Γ*_score_ for the newly generated peptide sequence draped on the backbone scaffold is evaluated.

(5) *MC Metropolis algorithm*: The MC Metropolis algorithm is used to accept or reject new trial peptides.

More details regarding the PepAD algorithm and $\Gamma_{\text{score}}$ can be found in our previous work (Xiao, Robang, et al., 2022). The development of the PepAD algorithm has been inspired by our previous work on designing peptides that bind to biomolecular targets using a *Pep*tide *B*inding *D*esign algorithm (Sarma et al., 2022; Sarma et al., 2023; Xiao, Kilgore, et al., 2022; Xiao, Sarma, et al., 2022).

### 4.2 DMD simulation and PRIME20 model

DMD simulations with the PRIME20 force field have been used to study the fibrilization kinetics of designed peptides by the Hall group. DMD is a fast alternative to traditional molecular dynamics simulations in which the interaction between two particles is modeled with a discontinuous potential, such as hard‐sphere, square‐well, or square‐shoulder potentials. The PRIME20 model is an implicit‐solvent coarse‐grained protein force field developed in the Hall group that was specifically designed for simulating peptide aggregation with DMD. In the PRIME20 model, each amino acid is represented by three backbone spheres (NH–, C_α_H–, and CO–) and one side chain sphere (R–). Each side chain of the 20 natural amino acids is assigned a unique size, atomic mass, and C_α_–R bond length. Details of the DMD simulations and PRIME20 model are described in our earlier work (Bunce et al., 2021; Cheon et al., 2010; Nguyen & Hall, 2004; Wagoner et al., 2012; Wang et al., 2016, 2017, 2018, 2019).

In this work, DMD/PRIME20 simulations of the PepAD‐generated peptides (PP1–PP8) were conducted at *T* = 296, 303, and 310 K for 5 μs for the preliminary screen. The temperature at which a peptide showed highest fibril formation was studied extensively by simulating that peptide for three runs, each at 12 μs. For each *in silico* peptide system, 48 peptides are placed into a cubic box with edge lengths of 200.0 Å, to achieve a peptide concentration ~ 10 mM. In each run, the peptide system starts from a random coil state. The DMD simulations were carried out in the canonical ensemble. The Andersen thermostat is implemented to maintain the simulation system at the desired temperature. Snapshots of the final simulated structures are obtained using the VMD 1.9.3 software.

### 4.3 Experimental assessment of self‐assembly

The sequences output by PepAD were experimentally tested by negative‐stain TEM, FTIR, ThT fluorescence, and CD using the methods we detailed previously (Collier & Messersmith, 2003). All peptides were imaged using negative‐stain TEM at a peptide concentration of 1 mg/mL in deionized water (DI water). FTIR measurements were conducted after a minimum of 72 h post assembly at a concentration of 10 mM in DI water. Additionally, we performed CD experiments at a concentration of 0.2 mM in DI water. ThT Fluorescence experiments were at a peptide concentration of 2 mM over an assembly period of 72 h.

## AUTHOR CONTRIBUTIONS

**Sudeep Sarma:** Investigation; writing – original draft; writing – review and editing; visualization; software; formal analysis; data curation; methodology; validation; conceptualization. **Tarunya Rao Sudarshan:** Investigation; writing – original draft; writing – review and editing; visualization; validation; methodology; formal analysis; data curation. **Van Nguyen:** Methodology; validation; data curation. **Alicia S. Robang:** Methodology; validation; data curation; formal analysis; conceptualization. **Xingqing Xiao:** Methodology; validation; software. **Justin V. Le:** Methodology; validation; data curation; formal analysis. **Michael E. Helmicki:** Methodology; validation; formal analysis; data curation. **Anant K. Paravastu:** Conceptualization; investigation; funding acquisition; writing – review and editing; project administration; resources; supervision. **Carol K. Hall:** Conceptualization; investigation; funding acquisition; writing – review and editing; project administration; supervision; resources.

## CONFLICT OF INTEREST STATEMENT

The authors declare that they have no known competing interests that could have appeared to influence the work reported in this paper.

## Supporting information


**Data S1:** Supporting Information

## Data Availability

Data is available in the manuscript and/or supporting information. The PDB files of starting conformations (Conf‐1, Conf‐2, and Conf‐3) and PepAD output files of PP1–PP8, PDB output files and beta‐sheet content from DMD/PRIME20 simulations of PP1‐PP8 and analysis codes are available at: https://github.com/CarolHall-NCSU-CBE/Parallel-self-assembling-peptides.
