# Supplementary material for: Design of parallel 𝛽‐sheet nanofibrils using Monte Carlo search, coarse‐grained simulations, and experimental testing
Source: Protein Sci. 2024 Jul 22;33(8):e5102. doi: 10.1002/pro.5102 (PMC11261811; doi:10.1002/pro.5102)
Supplement: Supplementary file 1 — Data S1: Supporting Information [file PRO-33-e5102-s001.docx]

**Supporting Information for Publication**

**Design of parallel 𝛽-sheet nanofibrils using Monte-Carlo search, coarse-grained simulations, and experimental testing**

Sudeep Sarma^1†^, Tarunya Rao Sudarshan^2†^, Van Nguyen^2^, Alicia S. Robang^2^, Xingqing Xiao^1,3^, Justin V. Le^2^, Michael E. Helmicki^2^, Anant K. Paravastu^2^, Carol K. Hall^1^

1. Department of Chemical and Biomolecular Engineering, North Carolina State University, Raleigh, NC 27695-7905, United States
2. Department of Chemical and Biomolecular Engineering, Georgia Institute of Technology, Atlanta, GA 30332, United States
3. Current address: Department of Chemistry, School of Chemistry and Chemical Engineering, Hainan University, Haikou City, Hainan Province 570228, P. R. China

^†^These authors contributed equally to the manuscript

*Corresponding Author

Email: [hall@ncsu.edu](mailto:hall@ncsu.edu)

+Co-Corresponding Author

Email: anant.paravastu@chbe.gatech.edu

**Supplementary Figures**


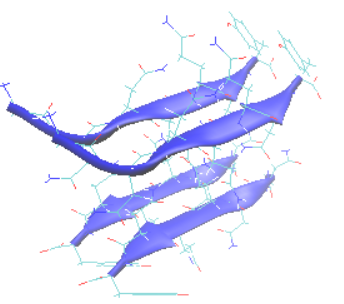


**Figure S1.** Class 1 cross-𝛽 spine containing 4 GNNQQNY peptides.


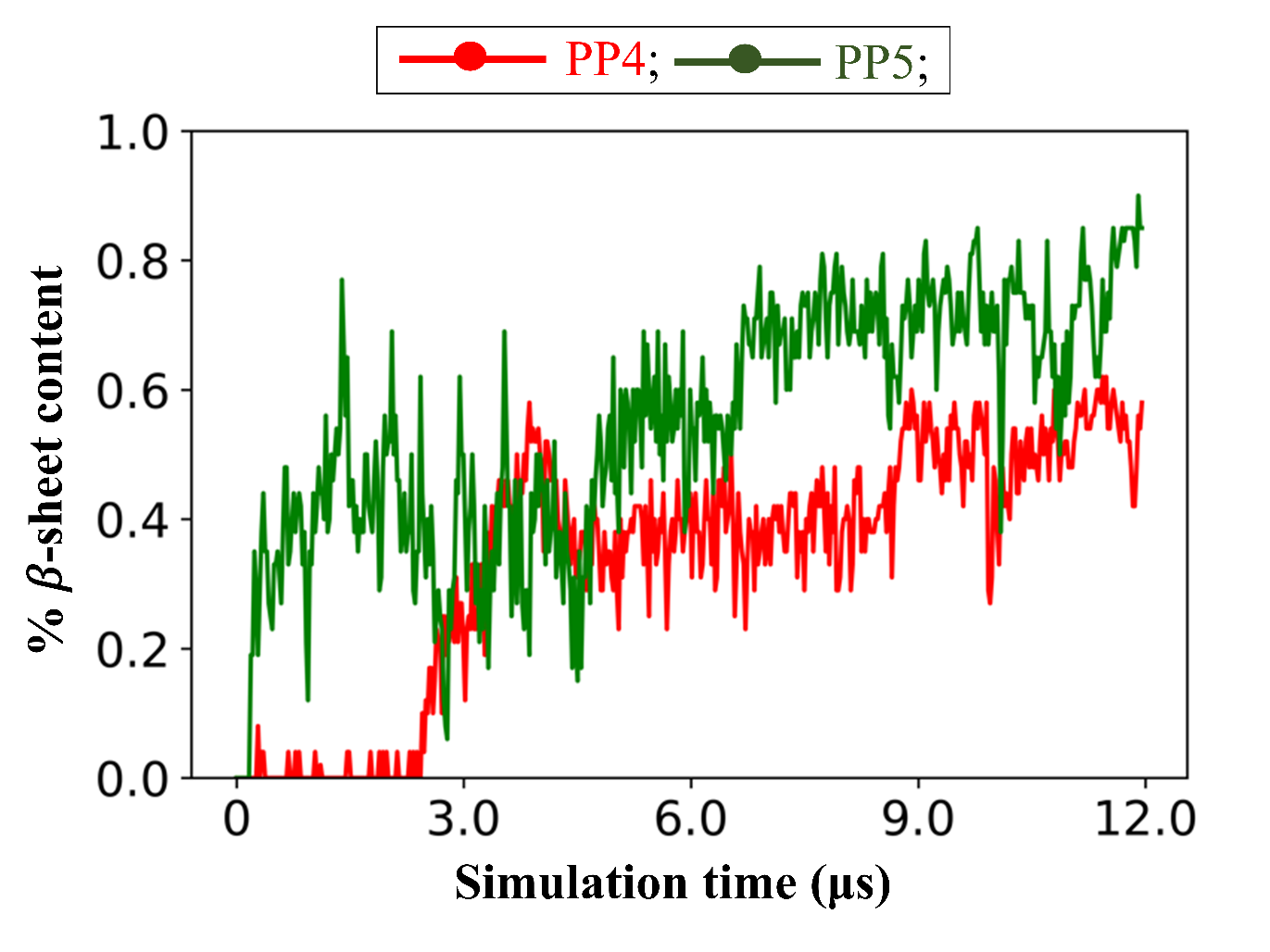


**Figure S2.** Plot of 𝛽-sheet content v/s simulation time describes the self-aggregation kinetics of peptide PP4: GAIDWVK and PP5: ADKVMFV.


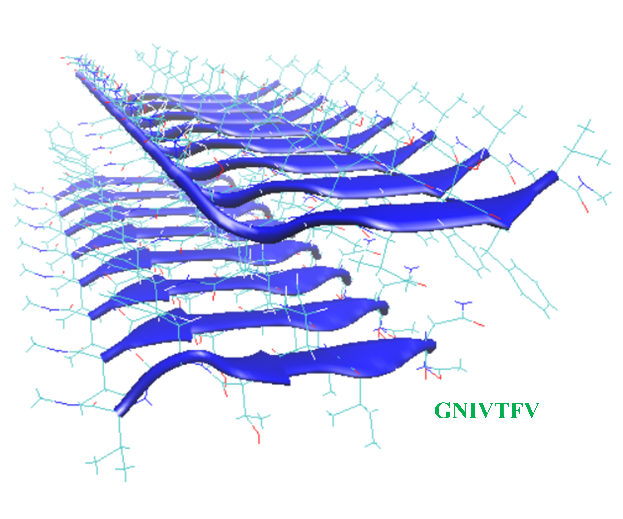


**Figure S3.** Class 1 cross-𝛽 spine structure containing 16 GNIVTFV (PP2) peptides.

**
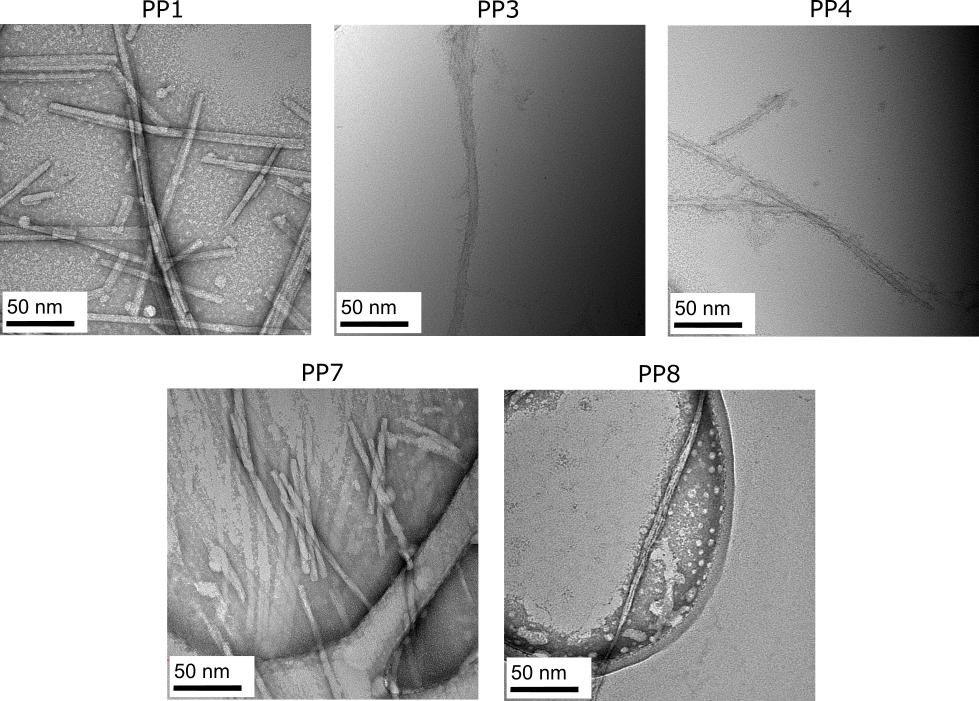
**

**Figure S4**. Transmission electron micrographs of negatively stained samples prepared one day following peptide dissolution at 1 mg/ml.

*
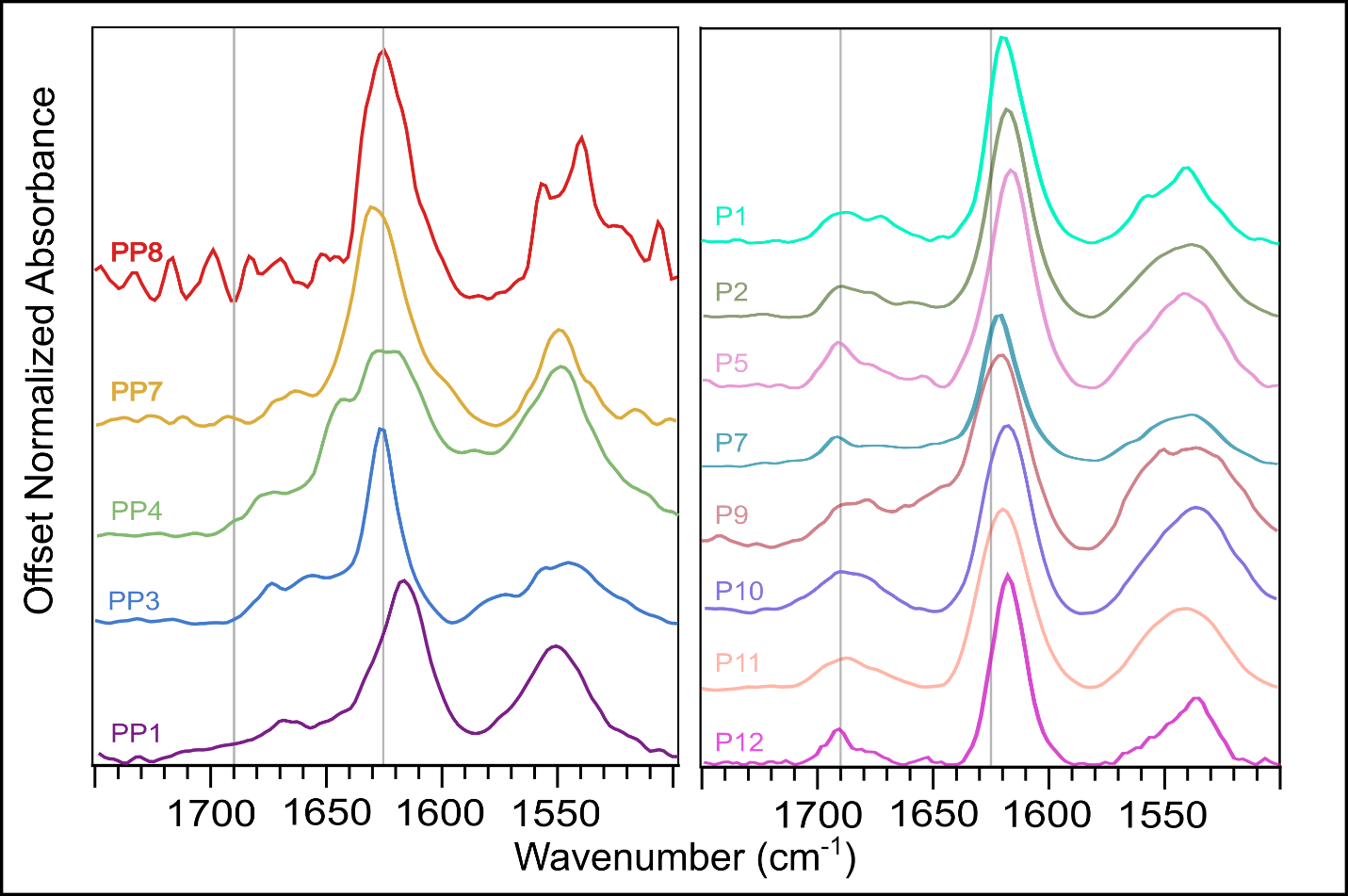
*

Figure S5: FTIR Spectra (smoothed and baseline corrected) for both current and previous study (left and right panels, respectively). The spectra in the left panel correspond to peptides designed to assemble into parallel β-sheets, whereas the spectra in the right panel were designed in our previous study to assemble into antiparallel β-sheets. Gray lines depict 1690 cm^-1^ and 1625 cm^-1^.


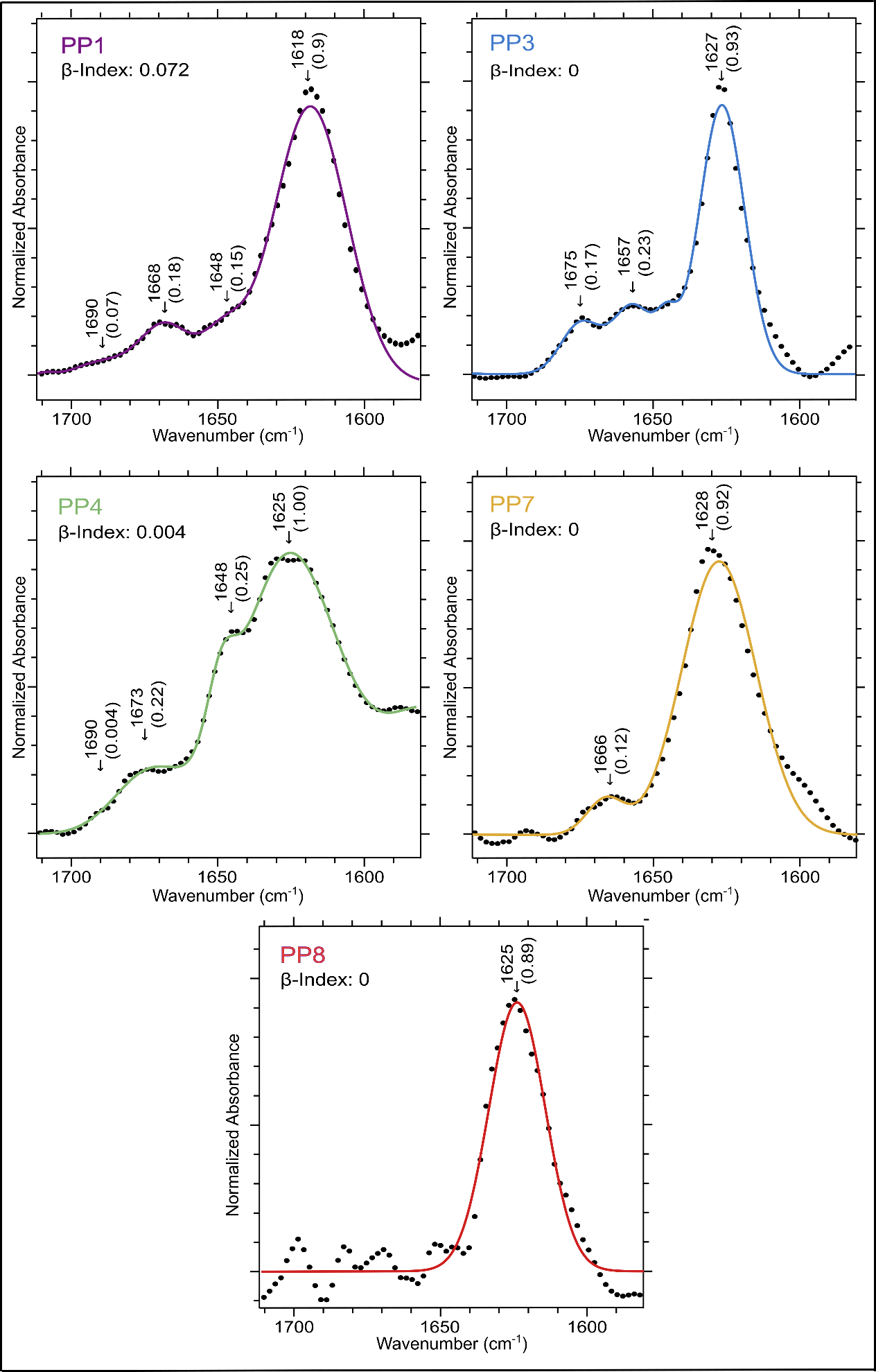


Figure S6: Gaussian Fitting for FTIR spectra for set of peptides designed to be parallel β-sheets (Present study)


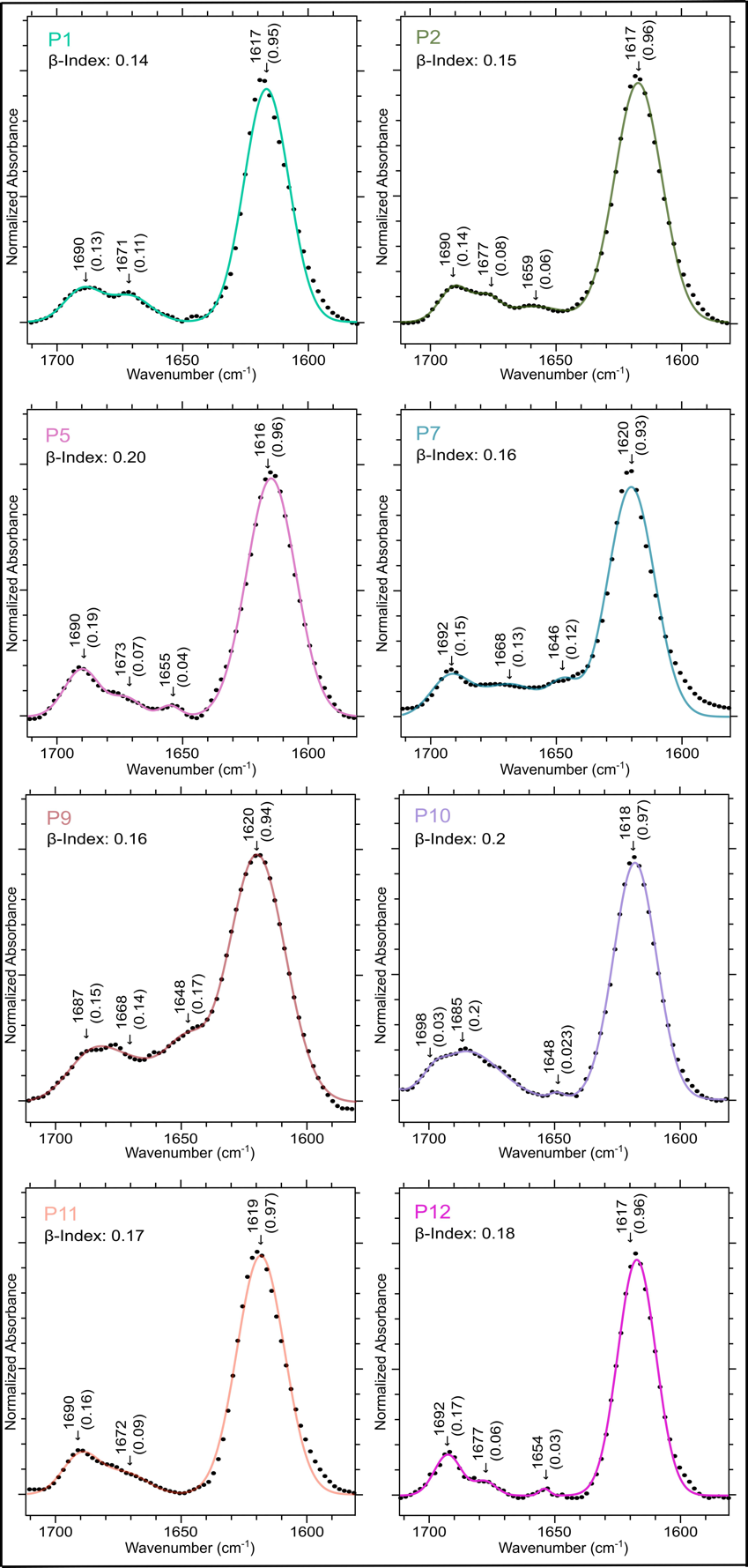


Figure S7: Gaussian Fitting for FTIR spectra for set of peptides designed to be antiparallel β-sheets^1^.

**
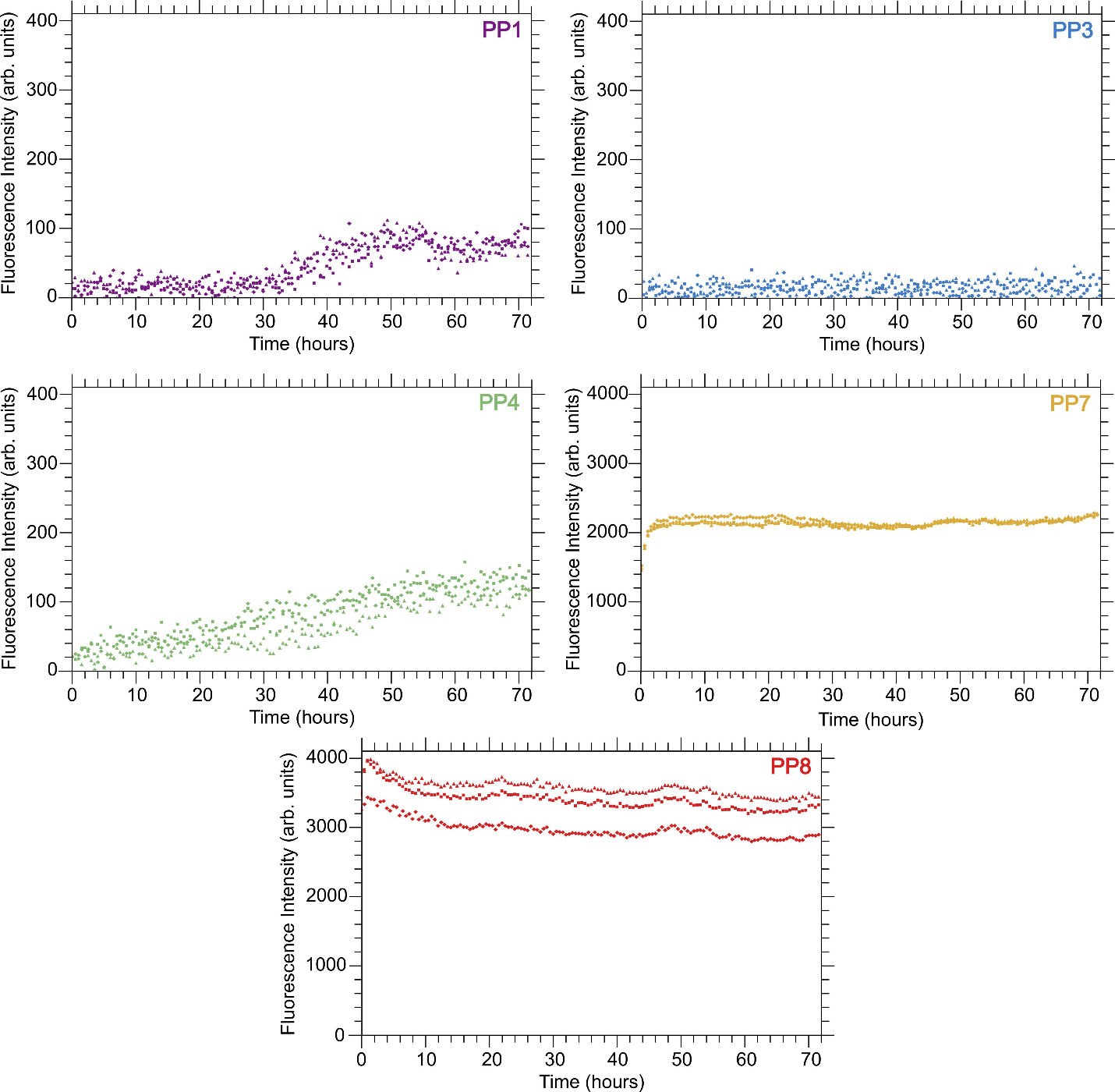
**

**Figure S8.** ThT fluorescence replicate curves for each of the five peptides predicted to aggregate.

**Peptide Synthesis**

Peptides (PP1, PP2, PP3, PP4, PP7 and PP8) were purchased from CPC Scientific, Inc. (Sunnyvale, CA). The peptides received at > 95% purity were used in experiments without modification.

**Thioflavin-T fluorescence**

Thioflavin-T (ThT) measurements were conducted using the procedure from our previous work^1^. Peptides were dissolved to a concentration of 2mM total peptide, 0.08mg/mL ThT, and 1X PBS before being added to a black 96-well plate (Thermo Scientific Nunc). Peptide samples were analyzed using a BioTek Synergy H4 Microplate Reader (excitation 450nm, emission 482nm, slit bandwidth 9nm), and fluorescence intensity was recorded over 72h. We performed ThT fluorescence measurements in triplicate, with the means of the samples reported in the main text.

**Fourier Transform Infrared Spectroscopy**

Fourier transform infrared spectroscopy (FTIR) was performed using protocol developed in our previous study^1^. These measurements were conducted on Thermo Scientific Nicolet 6700 spectrometer with an attenuated total reflection (ATR) accessory. Peptide solutions prepared at 10mM were spotted onto the ATR accessory, and an average over 64 scans was collected after an assembly period of 72h. As PP3 and PP4 assembled slower as compared to rest of peptides, FTIR scans were collected after 6 days of assembly. To calculate the β-index for each FTIR spectrum, we applied a moving average smoothing filter using Mathematica’s built-in “MovingAverage” function. We set this function to average 4 adjacent points for each point in the spectrum. We then used Mathematica’s built-in “EstimatedBackground” function to correct the baseline of each FTIR spectrum. We set this function to preserve spectral features with line widths up to 50 cm^-1^. Figure S5 shows the FTIR spectra after smoothing and baseline correction. Finally, we performed nonlinear regression of FTIR spectra to sums of Gaussian peaks at positions indicated in Figures S6 and S7. We calculated the β-index as the ratio of peak heights for the peak at 1690 cm^-1^ and the peak near 1620 cm^-1^. When we observed no discernable peak at 1690 cm^-1^, we report a β-index of 0 for the peptide.

FTIR unlike the other experimental techniques, can not only detect presence of β-sheets, but also differentiate between antiparallel and parallel organization of β-sheets. A low frequency, high intensity peak at ~1620cm^-1^ is attributed to β-sheets whereas a weaker intensity, high frequency peak at 1690cm^-1^ indicates an antiparallel organization of β-sheets^3,4^. Intuitively, the absence of this higher frequency peak is attributed to parallel β-sheets.

**Transmission Electron Microscopy**

Peptide solutions with DI water at 1mg/ml were prepared and assembled for minimum of 24h before measurements. Transmission Electron Microscopy (TEM) was conducted with a protocol like our previous work^1^. As PP3 and PP4 assembled slowly, an assembly period of 6 days was employed prior to TEM measurements.

**Circular Dichroism Spectroscopy**

Peptides were dissolved in water at a concentration of 0.2mM in 1X PBS. Subsequent to dissolution, concentration was verified using UV/Vis absorbance. Following a 2h assembly period, CD was measured at room temperature with a Chirascan^TM^-plus spectrometer (Applied Photophysics, Ltd.), following baseline correction with DI water without peptide. Quartz cuvettes with a 0.1mm path length were used. The protocol established for our previous study was employed to conduct measurements.

**REFERENCES**

1. Xiao X, Robang AS, Sarma S, et al. Sequence patterns and signatures: Computational and experimental discovery of amyloid-forming peptides. *PNAS Nexus*. 2022;1(5). doi:10.1093/pnasnexus/pgac263.
2. Micsonai A, Wien F, Bulyáki É, et al. BeStSel: a web server for accurate protein secondary structure prediction and fold recognition from the circular dichroism spectra. *Nucleic Acids Res.* 2018;46(W1). doi: 10.1093/nar/gky497.
3. Cerf E, Sarroukh R, Tamamizu-Kato S, et al. Antiparallel β-sheet: A signature structure of the oligomeric amyloid β-peptide. *Biochem J*. 2009;421(3). doi:10.1042/BJ20090379
4. Sarroukh R, Goormaghtigh E, Ruysschaert JM, Raussens V. ATR-FTIR: A “rejuvenated” tool to investigate amyloid proteins. *Biochim Biophys Acta - Biomembr*. 2013;1828(10). doi:10.1016/j.bbamem.2013.04.012
